# Identification of molecular subgroups and establishment of risk model based on the response to oxidative stress to predict overall survival of patients with lung adenocarcinoma

**DOI:** 10.1186/s40001-023-01290-5

**Published:** 2023-09-09

**Authors:** Linzhuang Liu, Qinghua Hou, Baorong Chen, Xiyi Lai, Hanwen Wang, Haozhen Liu, Liusheng Wu, Sheng Liu, Kelin Luo, Jixian Liu

**Affiliations:** 1 Peking University Shenzhen Hospital, Clinical College of Anhui Medical University, Shenzhen, 518036 Guangdong China; 2 Department of Thoracic Surgery, Peking University Shenzhen Hospital, Shenzhen, 518036 Guangdong China

**Keywords:** Lung adenocarcinoma, xCell, Oxidative stress, Immunotherapy, Risk model

## Abstract

**Objective:**

Oxidative stress is associated with the occurrence and development of lung cancer. However, the specific association between lung cancer and oxidative stress is unclear. This study aimed to investigate the role of oxidative stress in the progression and prognosis of lung adenocarcinoma (LUAD).

**Methods:**

The gene expression profiles and corresponding clinical information were collected from GEO and TCGA databases. Differentially expressed oxidative stress-related genes (OSRGs) were identified between normal and tumor samples. Consensus clustering was applied to identify oxidative stress-related molecular subgroups. Functional enrichment analysis, GSEA, and GSVA were performed to investigate the potential mechanisms. xCell was used to assess the immune status of the subgroups. A risk model was developed by the LASSO algorithm and validated using TCGA-LUAD, GSE13213, and GSE30219 datasets.

**Results:**

A total of 40 differentially expressed OSRGs and two oxidative stress-associated subgroups were identified. Enrichment analysis revealed that cell cycle-, inflammation- and oxidative stress-related pathways varied significantly in the two subgroups. Furthermore, a risk model was developed and validated based on the OSRGs, and findings indicated that the risk model exhibits good prediction and diagnosis values for LUAD patients.

**Conclusion:**

The risk model based on the oxidative stress could act as an effective prognostic tool for LUAD patients. Our findings provided novel genetic biomarkers for prognosis prediction and personalized clinical treatment for LUAD patients.

**Supplementary Information:**

The online version contains supplementary material available at 10.1186/s40001-023-01290-5.

## Introduction

Lung cancer remains the leading cause of cancer-related mortality and incidence, and it is the second most common tumor in the world [1]. Lung adenocarcinoma (LUAD) is the most common subtype of lung cancer, accounting for approximately 40% of all lung cancer [2]. And the proportion of LUAD is increasing year by year [3]. Despite some achievements have been made in the exploration of the pathogenesis and the development of novel therapies for LUAD patients. However, the 5-year survival rate for LUAD patients is still only 16% [4]. Immunotherapy and cancer immunology have gained popularity in cancer treatment in recent years [5]. It has been proposed to improve clinical outcomes in patients with LUAD [6, 7]. However, only about 20% of LUAD patients received satisfactory treatment [8]. Therefore, it is of great significance to understand the pathogenesis of lung cancer for the development of new targeted therapies to prolong the overall survival of LUAD patients.

Oxidative stress and the resulting oxidative injury are the primary contributors to the initiation and development of tumorigenesis [9]. Excessive reactive-oxygen species can cause genotoxicity and double-stranded DNA breaks, which result in genomic mutations and tumorigenesis [10, 11]. In addition, oxidative stress-related genes play an important role in the occurrence and development of several cancer types, including breast cancer, skin cancer, gastric cancer, cervical cancer, colorectal cancer, prostate cancer, etc. [12, 13]. Furthermore, oxidative stress is also associated with the prognosis of cancer patients. For example, a systematic review indicated lipid peroxidation might be related to overall survival after breast cancer diagnosis [14]. Oxidative stress contributes to premature mortality in colorectal cancer patients [15]. The bioinformatics approach identifies oxidative stress-associated genes that were significantly associated with the prognosis of gastric cancer [16]. Oxidative stress-related biomarkers are related to a poor prognosis in pancreatic cancer patients [17]. About 90% of lung cancer patients are directly associated with smoking. There is increasing evidence that smoking-evoked oxidative stress and reactive-oxygen species play an important role in cancer and inflammation [18, 19]. Pulmonary cancer initiation and development are associated with DNA oxidative injury and a series of oxidative stress-related pathways [20]. Recently, a study has indicated that the oxidative stress-related metabolic adaptation mediates radiation resistance in lung cancer cells [21]. Oxidative stress-associated lncRNAs are identified as potential markers to predict prognosis for LUAD patients [22]. Overall, these studies implied that oxidative stress is closely related to lung cancer development. However, the role of oxidative stress-related subtypes and genes in the prognostic prediction of lung cancer patients remains unclear, and the potential mechanism requires further investigation.

**Fig. 1:**
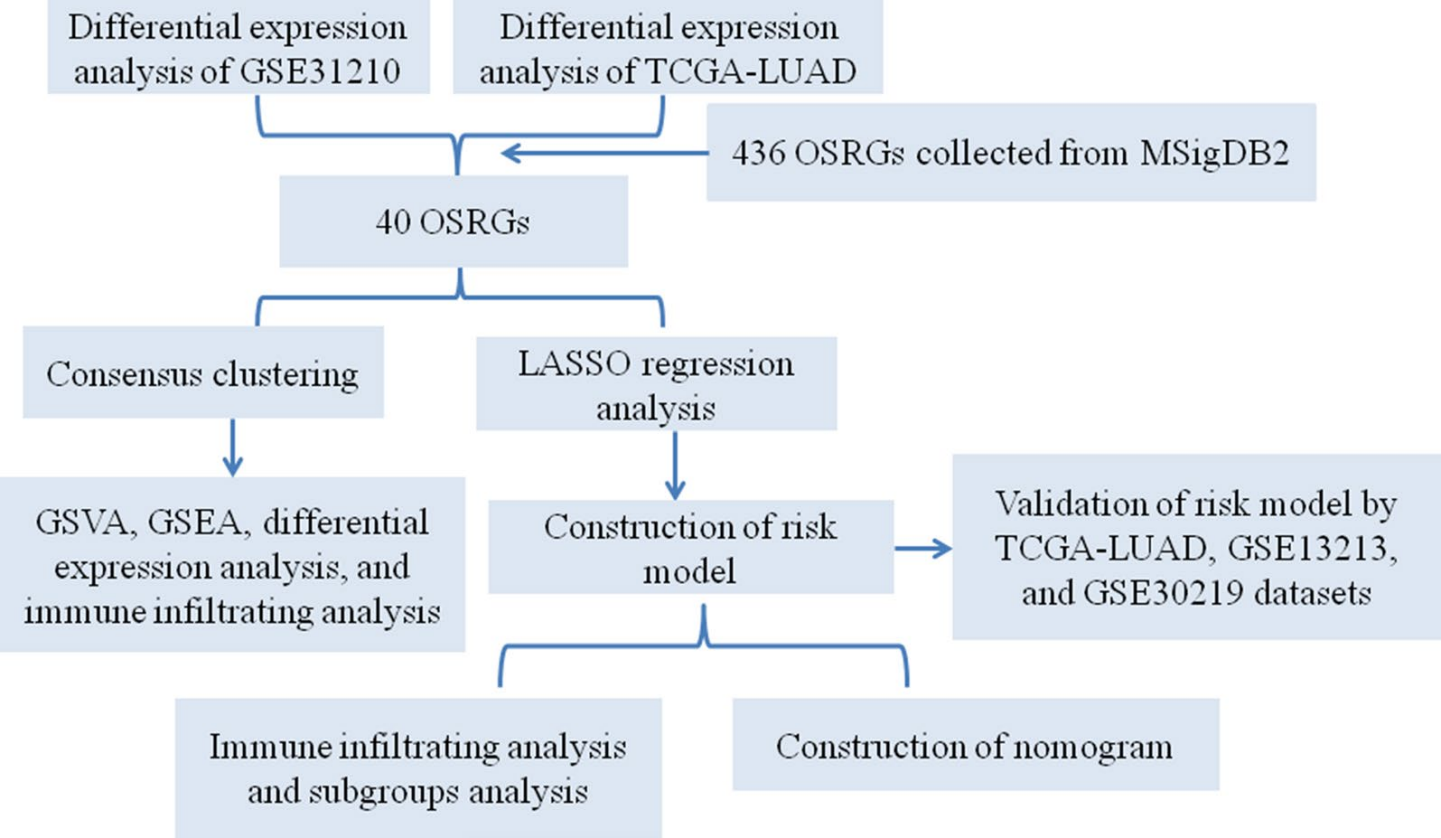
The flow chart presented the overall study design

In the recent years, with the development of tumor genomic sequencing technology, exploring novel tumor typing patterns by bioinformatics analysis provides a new way for tumor prognosis assessment and treatment [23–25]. In our study, the GEO and TCGA datasets were used to obtain the gene expression data of oxidative-related genes. Based on the oxidative stress-related genes, unsupervised clustering was applied to reveal the heterogeneity among LUAD patients and an oxidative stress-related risk model was developed and validated to predict the prognosis and immune microenvironment in LUAD patients. Figure 1 showed the overall framework of this research.

## Materials and methods

### Collection of datasets

Gene expression profiles and clinical information of LUAD patients were obtained from GEO and TCGA datasets. Only LUAD patients with pathologically confirmed and complete overall survival information were included in our study. Finally, a total of 226 LUAD cases obtained from the GSE31210 dataset, were used as the training cohort. Besides, we collected 522 cases from TCGA − LUAD, 79 cases from the GSE13213 dataset, and 57 cases from the GSE30219 dataset as public validation cohorts. For GEO datasets, we used “GEOquery” R package (2.34.0) to download the microarray data and the clinical information of LUAD cases. Then, the “sva” R package (3.18.0) and “limma” R package (3.26.9) were used to eliminate the batch effect and normalize data, respectively [26]. The average value of multi-probe genes was used as the gene expression value. For the TCGA − LUAD dataset, the “clusterProfiler” R package (3.10.1) was used to annotate the gene symbols [27]. The gene expression data were then handled by trimmed mean of M values normalization using the “edgeR” R package (2.6.12) [28]. When multiple expression values of the same gene, the average was used to represent the expression of that gene.

### Collection of differentially expressed oxidative stress-related genes (DEOSRGs) in LUAD patients

The differential expressed genes (DEGs) between the normal and tumor groups were identified with |log2 fold change (FC)|≥ 1 and p.adj < 0.05 using the “limma” package (3.26.9) of R [29]. The oxidative stress-related genes (OSRGs) were collected from the “GOBP RESPONSE TO OXIDATIVE STRESS” pathway in the Molecular Signatures Database. Then, the expression of DEOSRGs was presented as a heatmap using the “ComplexHeatmap” package (1.0.0) of R [30].

### Identification of oxidative stress-related molecular subtypes in LUAD patients

We performed consensus clustering based on the gene expression profile of DEOSRGs using the “ConsensusClusterPlus” package (1.2.0) of R [31]. We then repeated the optimal number of clusters between *k* = 2–10 and 1000 times to ensure the stability of the results. Then, the overall survival between the two subgroups was performed by Kaplan − Meier analysis.

### Identification of differentially expressed genes and characterization of immune cell infiltration between the subgroups

The differential expressed genes between the two subgroups were identified with |log2 fold change (FC)|≥ 1 and p.adj < 0.05 using the “limma” package (3.26.9) of R. The expression of the differential expressed genes was presented as a heatmap using the “ComplexHeatmap” package (1.0.0) of R. xCell is a novel genetic characterization method that enables comprehensive in silico assays to obtain 64 stromal and immune cells and compares them to cellular immune phenotypes [32]. Then, the relative abundance of 34 immune cell types was compared between the two subgroups by xCell algorithmic, and the results were presented as a histogram.

### Functional enrichment analysis

Enrichment analysis was carried out using the “clusterProfiler” package (3.10.1) in R to display the pathways in Metascape (v3.5.20230501) [33]. Gene set variation analysis (GSVA) of signal pathway changes between the two subgroups was performed using the “GSVA” package (1.30.0) in R [34], based on the “GO Biological Process” gene sets downloaded from the Molecular Signature Library. Gene set enrichment analysis (GSEA) was performed to evaluate whether there were significant differences in the expressed gene sets between the two subgroups during the enrichment of the MSigDB database (c2.cp.kegg.v7.4.symbols.gmt).

### Construction and assessment of the risk prognostic model based on DEOSRGs

Based on the DEOSRGs, we performed the least absolute shrinkage and selection operator (LASSO) regression analysis to construct a risk score model using the “glmnet” package (4.1.7) of R. A risk score was calculated for each LUAD patient in the training and verification cohorts based on the following calculation formula: risk score = expression value of EZH2×(− 0.0524) + expression value of ECT2×0.1908 + expression value of HYAL1×0.0371 + expression value of TLR4×(− 0.1355) + expression value of GPX8×0.1043 + expression value of GJB2×0.0722 + expression value of CDK1×0.005 + expression value of GPR37×0.1081 + expression value of GPX2×0.0203 + expression value of GPX3×(− 0.0332) [23, 24, 35]. Besides, we used TCGA-LUAD, GSE13213, and GSE30219 datasets to confirm the prognostic value of the risk score model. We also used the Kaplan − Meier method to determine the probability of survival between two risk score groups, and the area under the curve (ROC) was applied to determine the specificity and sensitivity of the risk score model. Cox regression was then used to determine whether risk score was a reliable predictor for LUAD patients. In addition, the “rms” R package (6.3.0) was used to generate a nomogram for the prediction of 3- and 5-year survival rates for LUAD patients.

### Quantitative real-time polymerase chain reaction (qRT-PCR)

In 2021–2022, ten pairs of cancerous tissue/peritumoral tissue were taken from lung cancer patients who had surgery at the Department of Thoracic Surgery, Peking University Shenzhen Hospital. The ethics committee of Peking University Shenzhen Hospital approved this research. The identification of LUAD patients was conducted using the following inclusion criteria: (1) LUAD patients diagnosed between 2021 and 2022 in China; (2) pathologic diagnosis of LUAD and (3) surgical treatment. The exclusion criteria were as follows: (1) any unknown clinical information and epidemiology; (2) age at diagnosis < 18; (3) presence of multiple primary cancers; (4) presence of other pathology; (5) neoadjuvant treatment. A total of 10 patients were enrolled. The characteristics of the patients are presented in Additional file 1: Table S1. With TRIzol reagent (Invitrogen, CA, USA), total RNA was taken out of the tissue. The reverse transcriptase (Invitrogen, CA, USA) was used to make cDNA. Relative expression of the gene was analyzed by SYBR green qRT-PCR assay (Sigma, MO, USA) on an ABI 7900HT System (Applied Biosystems). The primers used in the present study were presented in Additional file 1: Table S2.

### Statistical analysis

All analyses were performed in R software (3.4.1). The Wilcoxon’s test was used to compare between two groups. *p* < 0.05 was considered statistically significant.

## Results

### Identification of DEOSRGs in LUAD patients

**Fig. 2:**
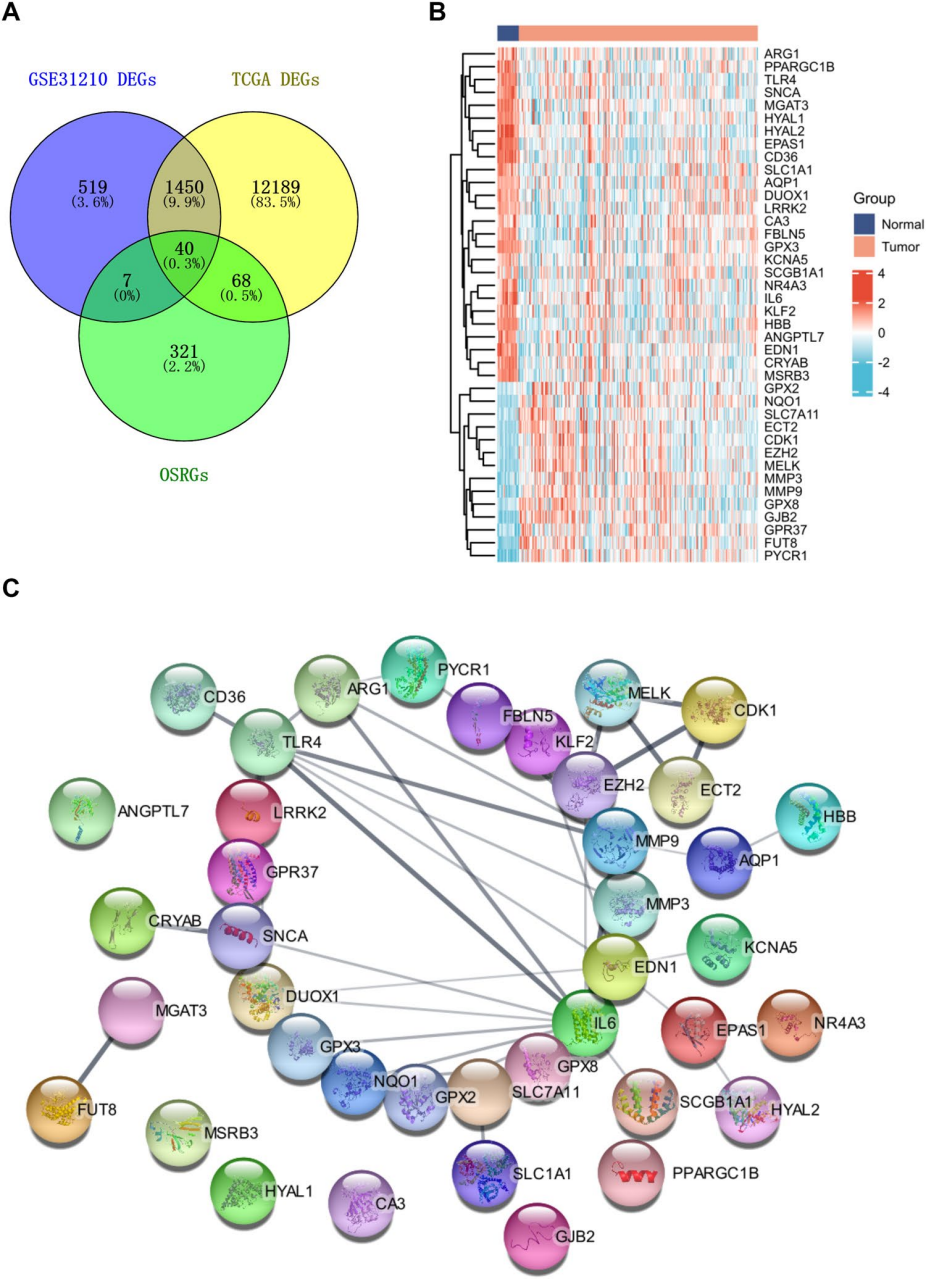
Identification of OSRGs in LUAD patients. **A** Venn diagram for the identification of DEOSRGs in GSE31210 and TCGA datasets. **B** The heatmap represented the 40 DEOSRGs expression pattern in the normal and LUAD groups. **C** PPI network among the 40 DEOSRGs

A total of 2016 (Additional file 2: Table S3) and 13,747 DEGs (Additional file 3: Table S4) were identified in GSE31210 and TCGA-LUAD databases, respectively. 436 OSRGs were collected from the Molecular Signatures Database. Then, 40 DEOSRGs were obtained by the intersection of DEGs and OSRGs (Fig. 2A). The expression pattern of DEOSRGs in normal and tumor samples was also analyzed. As showed in Fig. 2B, ARG1, PPARGC1B, TLR4, SNCA, MGAT3, HYAL1, HYAL2, EPAS1, CD36, SLC1A1, AQP1, DUOX1, LRRK2, CA3, FBLN5, GPX3, KCNA5, SCGB1A1, NR4A3, IL6, KLF2, HBB, ANGPTL7, EDN1, CRYAB, and MSRB3 were down-regulated in LUAD patients, while GPX2, NQO1, SLC7A11, ECT2, CDK1, EZH2, MELK, MMP3, MMP9, GPX8, GJB2, GPR37, FUT8, and PYCR1 were up-regulated in LUAD patients. The protein − protein interaction of 40 DEOSRGs was constructed by the STRING database to further exhibit the interconnections between those genes (Fig. 2C).

### Identification of two oxidative stress-related subgroups

**Fig. 3:**
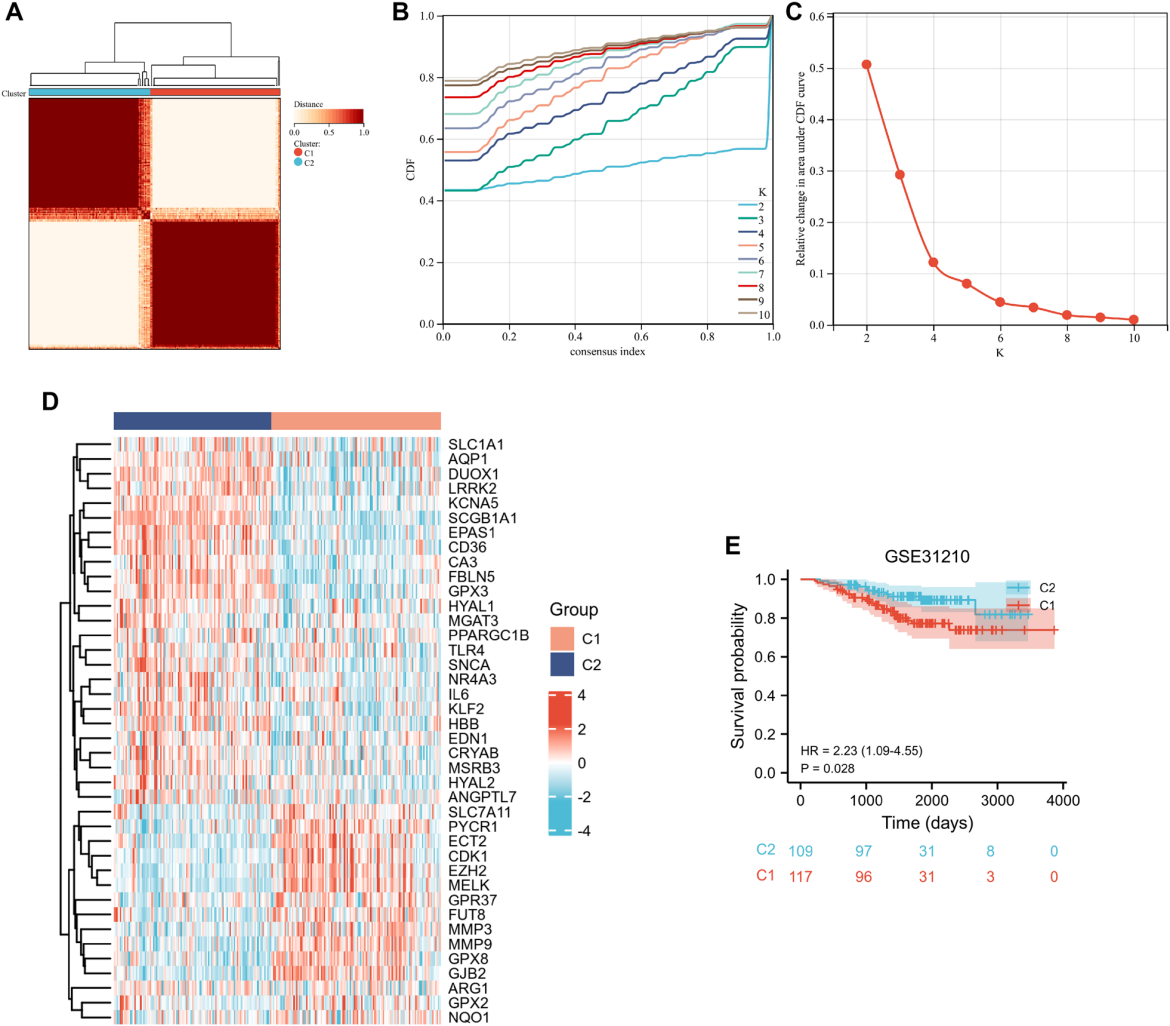
Identification of oxidative stress-related subgroups in the GSE31210 dataset using consensus clustering. **A**–**C** *K* = 2 was considered the optimal clustering stability. **D** The heatmap represented the 40 DEOSRGs expression pattern in the C1 and C2 subgroups. **E** Kaplan − Meier curves in the two subgroups

In the GSE31210 dataset, the LUAD patients were divided into two subgroups based on the 40 DEOSRGs by the consensus clustering analysis (Fig. 3A–C). Besides, Fig. 3D represented the expression pattern of DEOSRGs between the two subgroups, and significant expression differences were observed between the C1 and C2. In addition, LUAD patients in C1 exhibited adverse clinical outcomes compared with LUAD patients in C2 (*p* = 0.028, Fig. 3E). Our findings revealed that the DEOSRGs could divide LUAD patients into two subgroups with different survival rates.

### Identification of differentially expressed genes and characterization of immune cell infiltration between the subgroups

**Fig. 4:**
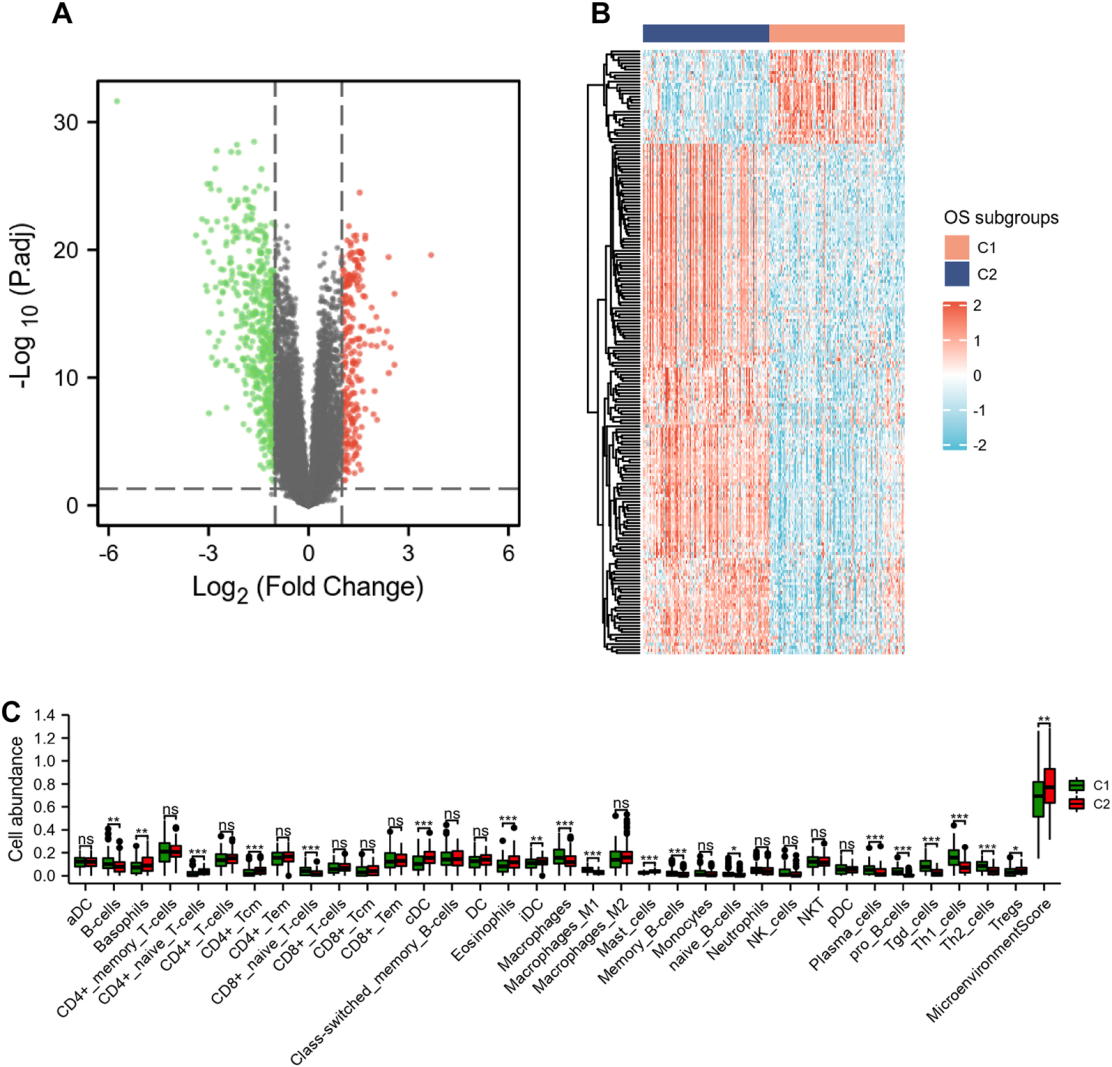
Identification of differentially expressed genes and characterization of immune cell infiltration between the subgroups. Volcano plot (**A**) and heatmap (**B**) present the distribution and expression of differentially expressed genes between the C1 and C2 subgroups. The green dots are the down-regulated genes and red dots are the up-regulated genes. **C** Box plots visualize the immune cell infiltration levels between the C1 and C2 subgroups. *p < 0.05, **p < 0.01, and ***p < 0.001

As shown in Fig. 4A, a total of 754 abnormal expressed genes were identified, including 239 up-regulated genes and 515 down-regulated genes in C1, as compared to C2. Figure 4B represented the expression pattern of the top 200 differentially expressed genes between C1 and C2. Furthermore, the xCell algorithm revealed that LUAD patients have different immune statuses and tumor immune microenvironments in the C1 and C2. As shown in Fig. 4C, the cell abundance of B cells, CD8+ naive T cells, macrophages, macrophages M1, memory B cells, naive B cells, pro B cells, Tgd cells, Th1 cells, and Th2 cells in C1 subgroup was higher than C2 subgroup (*p* < 0.05), while the cell abundance of basophils, CD4+ naive T cells, CD4+ Tcm, cDC, eosinophils, iDC, and mast cells in C2 subgroup was lower than C2 subgroup (*p* < 0.05).

### Functional enrichment analyses

**Fig. 5:**
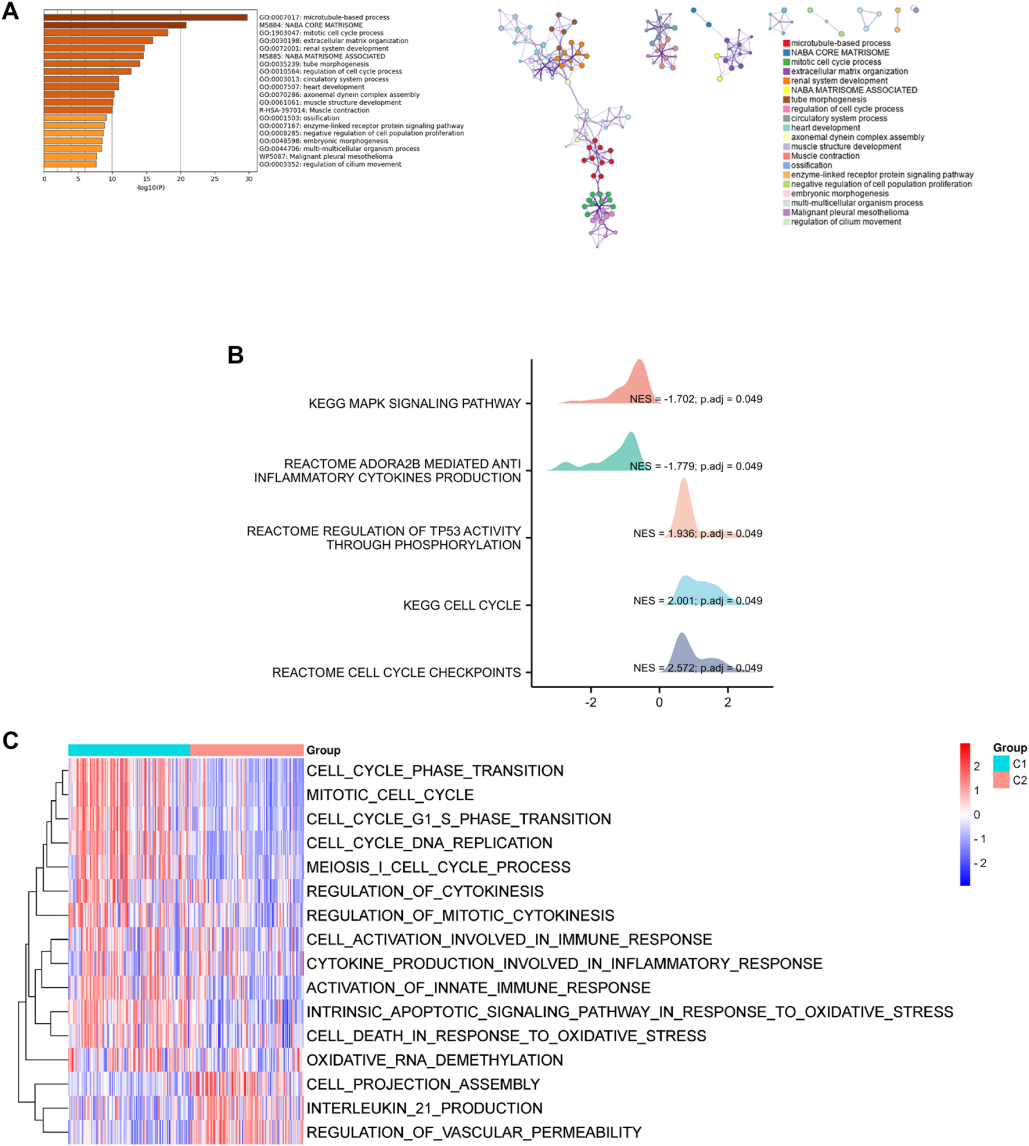
Functional enrichment analyses. **A** Bar diagram presented the potential pathways. **B** Multipeaked maps visualized the results of GSEA. **C** Heat map presented the differential signaling pathways between C1 and C2 subgroups

As shown in Fig. 5A, functional enrichment analysis indicated that differentially expressed genes between C1 and C2 were mainly enriched in cell cycle-related pathways, such as the mitotic cell cycle process, regulation of cell cycle process, and negative regulation of cell population proliferation, etc. In addition, we carried out GSEA and GSVA analyses to further assess the expression difference of potential pathways in two subgroups. As shown in Fig. 5B, GSEA results revealed that MAPK signaling pathway, ADORA2B-mediated anti-inflammatory cytokines production, regulation of TP53 activity through phosphorylation, cell cycle, and cell cycle checkpoints were differentially enriched pathways between two subgroups. In comparison to the C2 subgroup, cell cycle phase transition, mitotic cell cycle, cell cycle NDA replication, regulation of cytokinesis, cell activation involved in immune response, cytokine production involved in inflammatory response, activation of innate immune response, intrinsic apoptotic signaling pathway in response to oxidative stress, cell death in response to oxidative stress, and oxidative RNA demethylation were activated in C1 subgroup (Fig. 5C). These findings revealed that expression of oxidative stress-related was associated with dysregulation of immune and oxidative stress, which may be implicated in the poor prognosis of LUAD patients.

### Construction and validation of a prognostic risk score model for LUAD patients

**Fig. 6:**
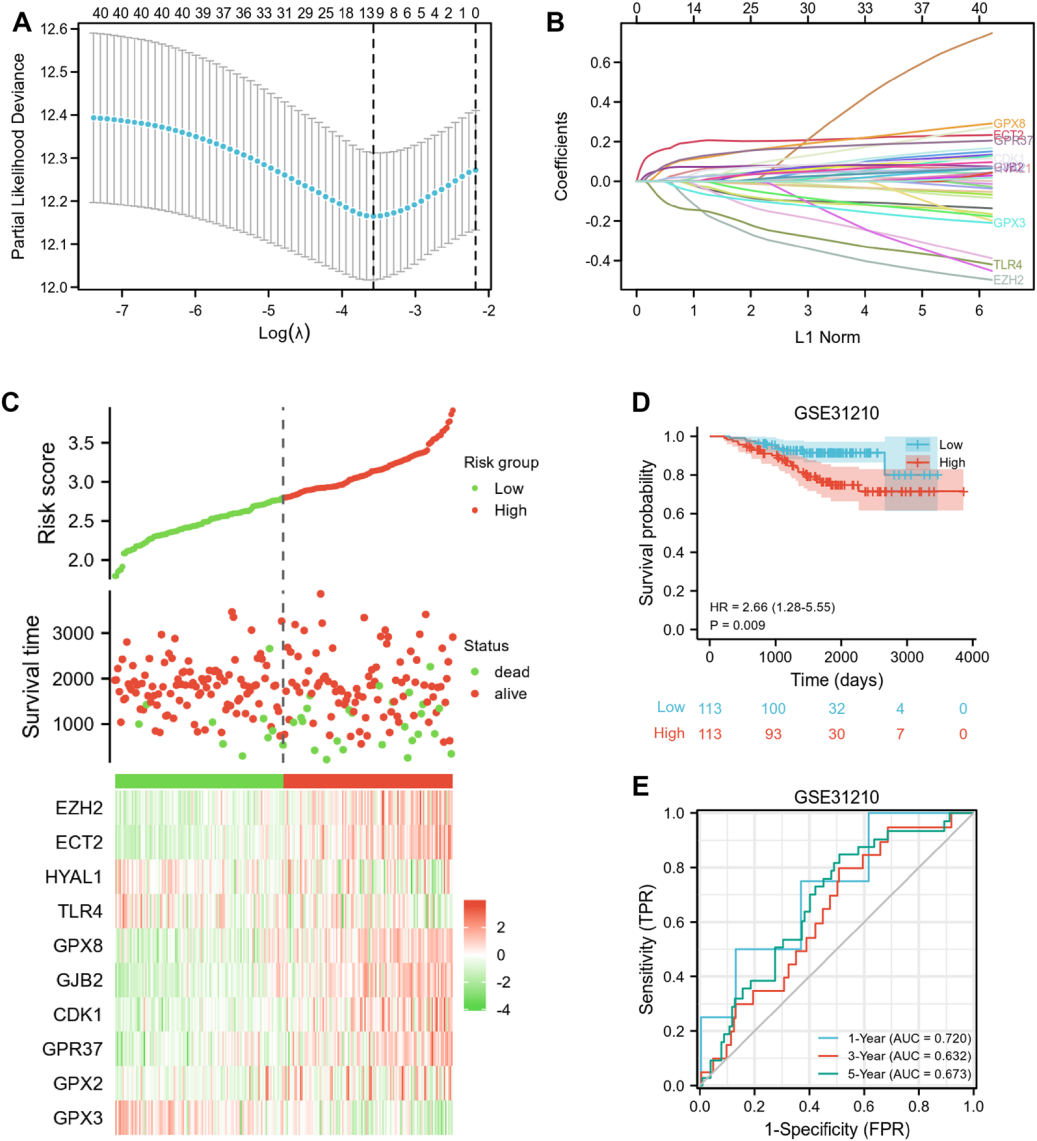
Development of risk score model based on 40 oxidative stress-related genes. **A**–**B** The LASSO analysis identified 10 prognostic genes for LUAD patients. **C** The distribution of survival status and the expression of 10 prognostic genes between the low and high-risk groups. **D** Kaplan − Meier curves for the low and high-risk groups in the GSE31210 dataset. **E** Time-dependent ROC curves of the risk score model

**Fig. 7:**
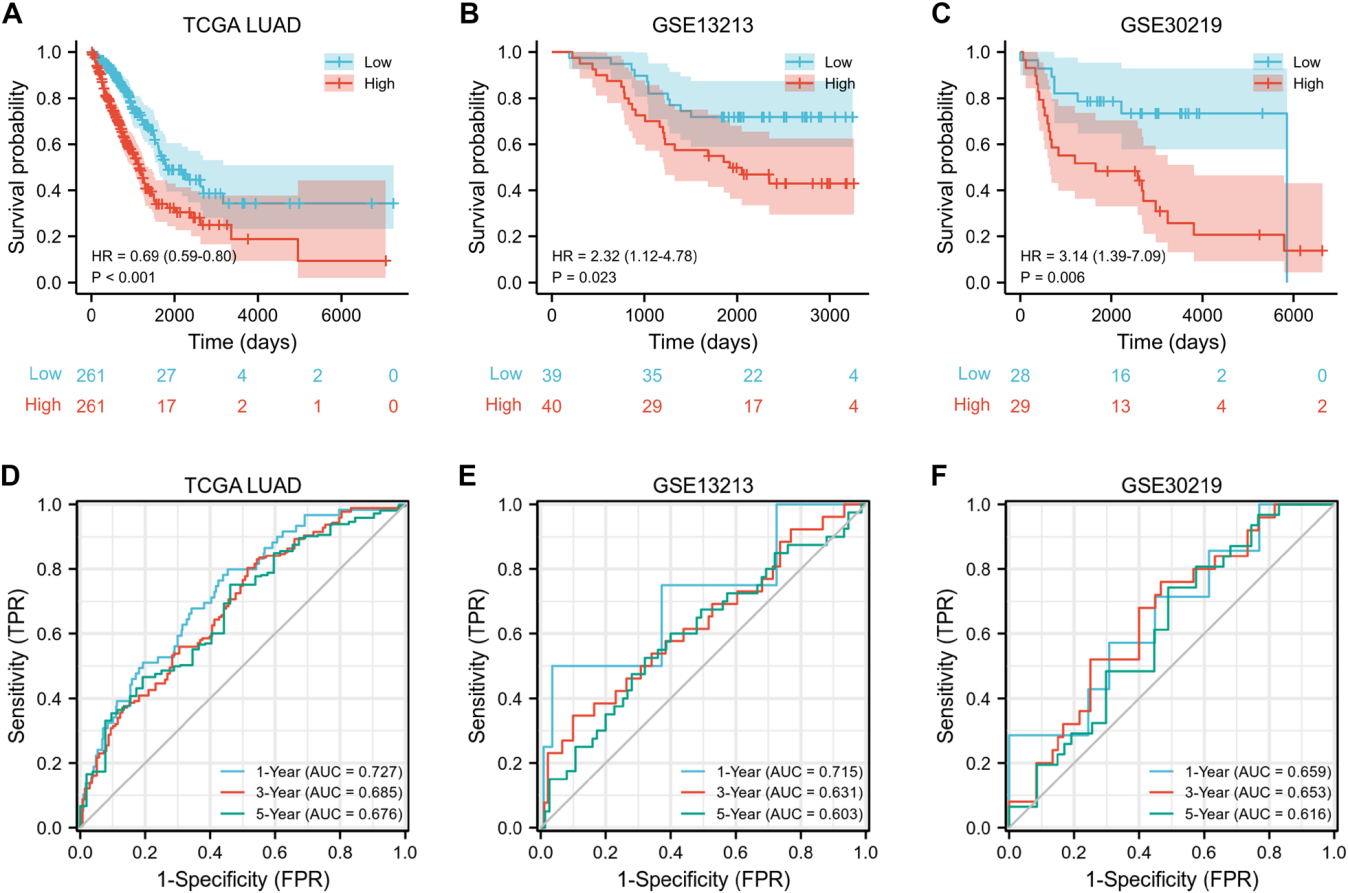
Validation of the risk score model by the independent datasets. Kaplan − Meier curves for the low and high-risk groups in the TCGA-LUAD (**A**), GSE13213 (**B**), and GSE30219 (**C**) datasets. Time-dependent ROC curves of the risk score model in the TCGA-LUAD (**D**), GSE13213 (**E**), and GSE30219 (**F**) datasets

We performed LASSO Cox regression analysis to develop an oxidative stress-related risk score model based on 40 DEOSRGs (Fig. 6A, B). Then, 10 DEOSRGs were selected for the construction of risk score model according to following calculation formula: expression value of EZH2× (− 0.0524) + expression value of ECT2×0.1908 + expression value of HYAL1×0.0371+ expression value of TLR4×(− 0.1355) + expression value of GPX8×0.1043 + expression value of GJB2×0.0722 + expression value of CDK1×0.005 + expression value of GPR37×0.1081 + expression value of GPX2×0.0203 + expression value of GPX3×(− 0.0332). As shown in Fig. 6C, we also found the number of dead statuses in the high-risk group was higher, when compared with those in the low-risk group. EZH2, ECT2, GPX8, GJB2, CDK1, GPR37, and GPX2 exhibited lower expression levels in the low-risk group, whereas HYAL1, TLR4, and GPX3 exhibited lower expression levels in the high-risk group. The high-risk score group exhibited a poor prognosis for LUAD patients (Fig. 6D, p = 0.009). In addition, the risk model presented certain sensitivity and specificity, with the area under the curve (AUC) values of 1, 3, and 5 years were 0.72, 0.632, and 0.673, respectively (Fig. 6E). The risk score model was also validated by external datasets. As shown in Fig. 7A–C, survival analysis indicated that the high-risk LUAD patients exhibited worse prognosis in TCGA-LUAD (*p* < 0.01), GSE13213 (*p* = 0.023), and GSE30219 (*p* = 0.006) datasets. Furthermore, the ROC analysis indicated that the risk score model had certain prediction accuracy for LUAD patients (Fig. 7D–F).

We also performed univariate and multivariate Cox analyses to evaluate the independence of the predictive value of the risk score. As presented in Table 1, we found that only risk score (HR = 3.298, *p* = 0.002) presented a good predictive ability of prognosis. In addition, multivariate Cox analysis revealed the risk score was an independent predictor for LUAD patients (HR = 2.111, *p* = 0.047).

**Table 1:** Univariate and multivariate analysis of risk score and clinical features

Characteristics	Total(N)	Univariate analysis		Multivariate analysis	
		Hazard ratio (95% CI)	*P* value	Hazard ratio (95% CI)	*P* value
Gender	226				
Male	105	Reference			
Female	121	0.658 (0.338–1.281)	0.219		
Age (years)	226	1.025 (0.977–1.075)	0.306		
Smoking status	226				
Never-smoker	115	Reference			
Ever-smoker	111	1.637 (0.837–3.201)	0.150		
Risk score	226	3.298 (1.553–7.006)	**0.002**	2.111 (0.950–4.692)	**0.047**

Bold indicates statistically significant differences

### Construction and verification of the nomogram

**Fig. 8:**
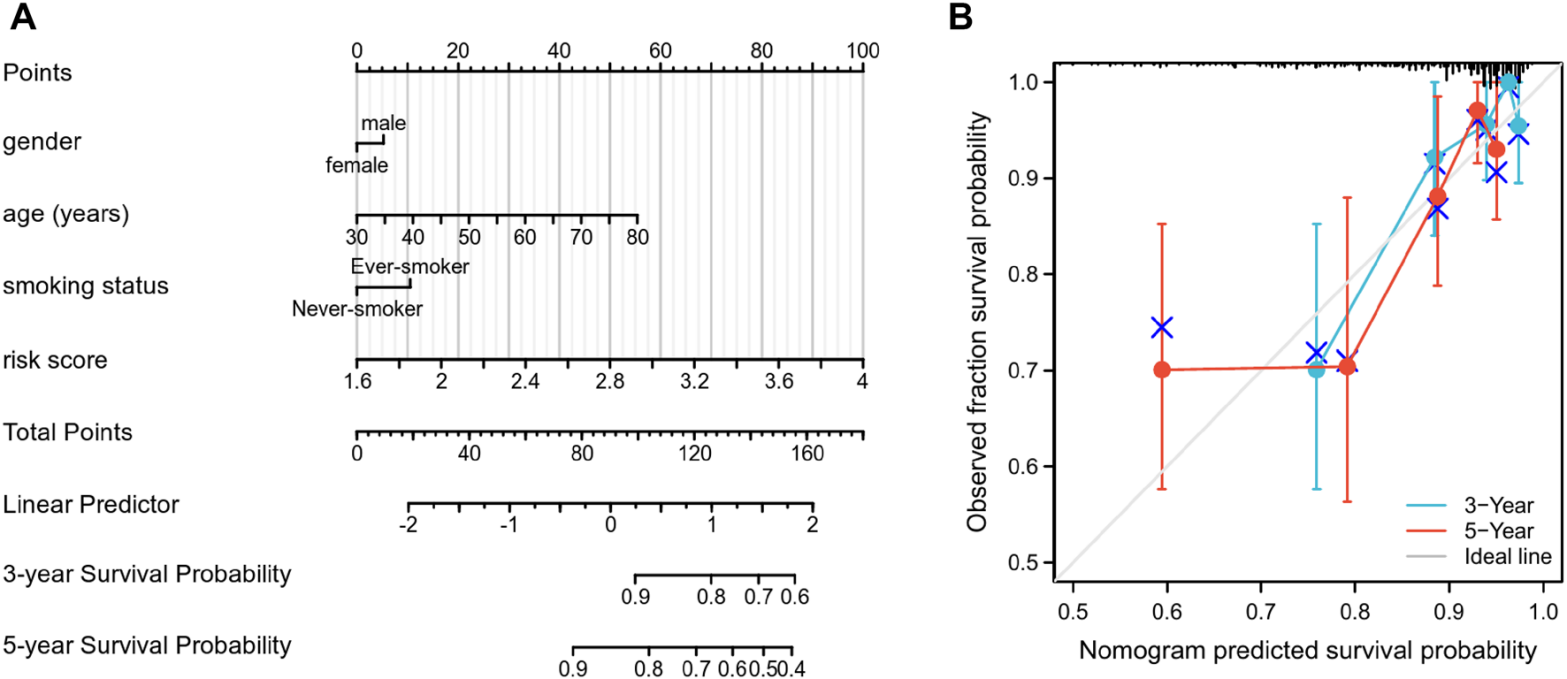
Construction and assessment of nomogram for the risk score model. **A** Nomogram for the prediction of the prognosis in LUAD patients. **B** Calibration for the 3- and 5-year overall survival in LUAD patients

As presented in Fig. 8A, a nomogram contained some important clinical features and a risk score was constructed. In addition, we also found the predicted and actual overall survival was consistent by drawing the calibration curve (Fig. 8B).

### Analysis of immune cell infiltration between the low- and high-risk score groups

**Fig. 9:**
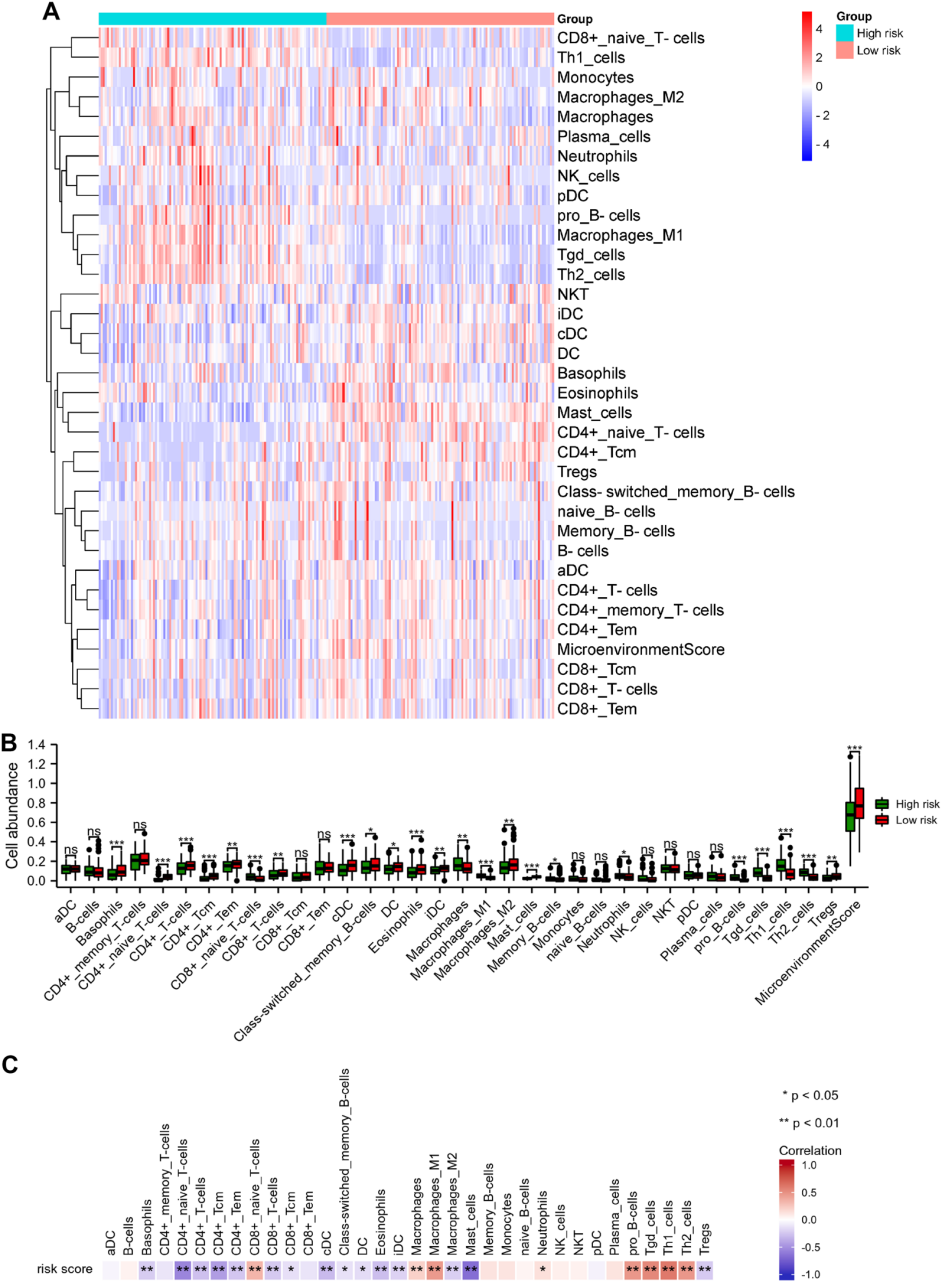
Immune cell infiltration analysis between the low and high-risk groups. **A** The heatmap visualized the differential levels of immune cells. **B** The histograms presented the differential levels of immune cells between low and high-risk groups. **C** The correlation analysis between 34 immune cells and risk score. *p < 0.05, **p < 0.01, and ***p < 0.001

We used the xCell algorithm to assess the difference of 34 immune cell types in the lung cancer microenvironment. The differential levels of immune cell infiltration between the two groups were presented in Fig. 9A, B. We found that the proportion of basophils, CD4+ naïve T cells, CD4+ T cells, CD4+ Tcm, CD4+ Tem, CD8+ T cells, cDC, class-switched memory B cells, DC, eosinophils, iDC, macrophages M2, mast cells, and Tregs in high-risk score group was lower than low-risk score group (*p* < 0.05), while the proportion of CD8+ naïve T cells, macrophages, macrophages M1, memory B cells, neutrophils, pro B cells, Tgd cells, Th1 cells, and Th2 cells in the high-risk score group was higher than low-risk score group (*p* < 0.05). In addition, the correlation between the risk score and immune cell infiltration was also assessed. As shown in Fig. 9C, risk score had a significant negative correlation with basophils, CD4+ naïve T cells, CD4+ T cells, CD4+ Tcm, CD4+ Tem, CD8+ T cells, CD8+ Tcm, cDC, class-switched memory B cells, DC, eosinophils, iDC, macrophages M2, mast cells, and Tregs. The risk score had a significant positive correlation with CD8+ naïve T cells, macrophages, macrophages M1, neutrophils, pro B cells, Tgd cells, Th1 cells, and Th2 cells.

**Fig. 10:**
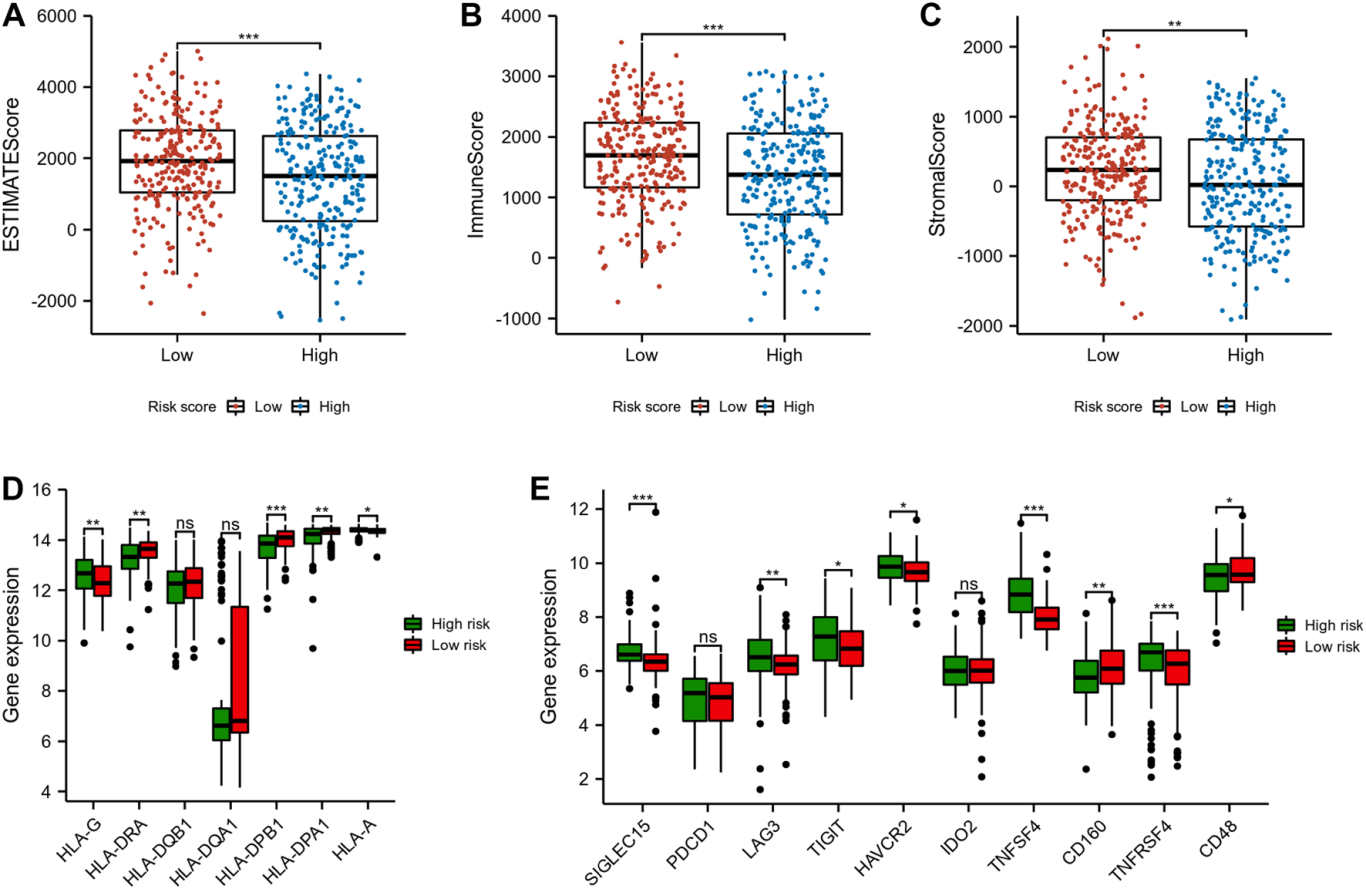
Immune analyses between low and high-risk score groups. The ESTIMATE algorithm was used to calculate the ESTIMATE score (**A**), immune score (**B**), and stromal score (**C**). The histograms presented the differential levels of HLA genes (**D**) and immune checkpoints genes (**E**) between low and high-risk groups. *p < 0.05, **p < 0.01, and ***p < 0.001

As shown in Fig. 10A–C, the results of the ESTIMATE algorithm showed that high-risk score group had a lower ESTIMATE score, immune score, and stromal score, as compared with the low-risk score group (*p* < 0.01). Furthermore, we also found some human leukocyte antigen (HLA) genes and immune checkpoints genes were down-regulated in the high-risk score group, including HLA-DRA, HLA-DPB1, HLA-DPA1, CD160, and CD48 (Figs. 10D, E). These findings implied that the prognosis of different LUAD risk score subtypes might be influenced by the tumor immune microenvironment.

### Immune cell infiltration was associated with poor prognosis in LUAD patients

In the present study, the association between survival time and immune cell infiltration was also analyzed. As presented in Additional file 1: Figures S1A–F, our results indicated that high levels of macrophages, macrophages M1, Tgd cells, pro B cells, Th1 cells, and Th2 cells were associated with shorter survival times (*p* < 0.05).

### Analysis of the prognostic value of risk model-related genes

As shown in Additional file 1: Figure S2, CDK1, ECT2, EZH2, GJB2, GPR37, GPX2, and GPX8 mRNA expression was up-regulated in the tumor group (*p* < 0.001), whereas GPX3, HYAL1, and TLR4 mRNA expression was down-regulated in the tumor group (*p* < 0.001) compared with those in the normal group. The Kaplan − Meier analysis indicated that high expression of CDK1, ECT2, GJB2, GPR37, GPX2, and GPX8 was associated with poor prognosis in LUAD patients; low expression of TLR4 was associated with poor prognosis in LUAD patients (Additional file 1: Figure S3). Therefore, CDK1, ECT2, GJB2, GPR37, GPX2, GPX8, and TLR4 were potential prognostic biomarkers for LUAD patients.

**Fig. 11:**
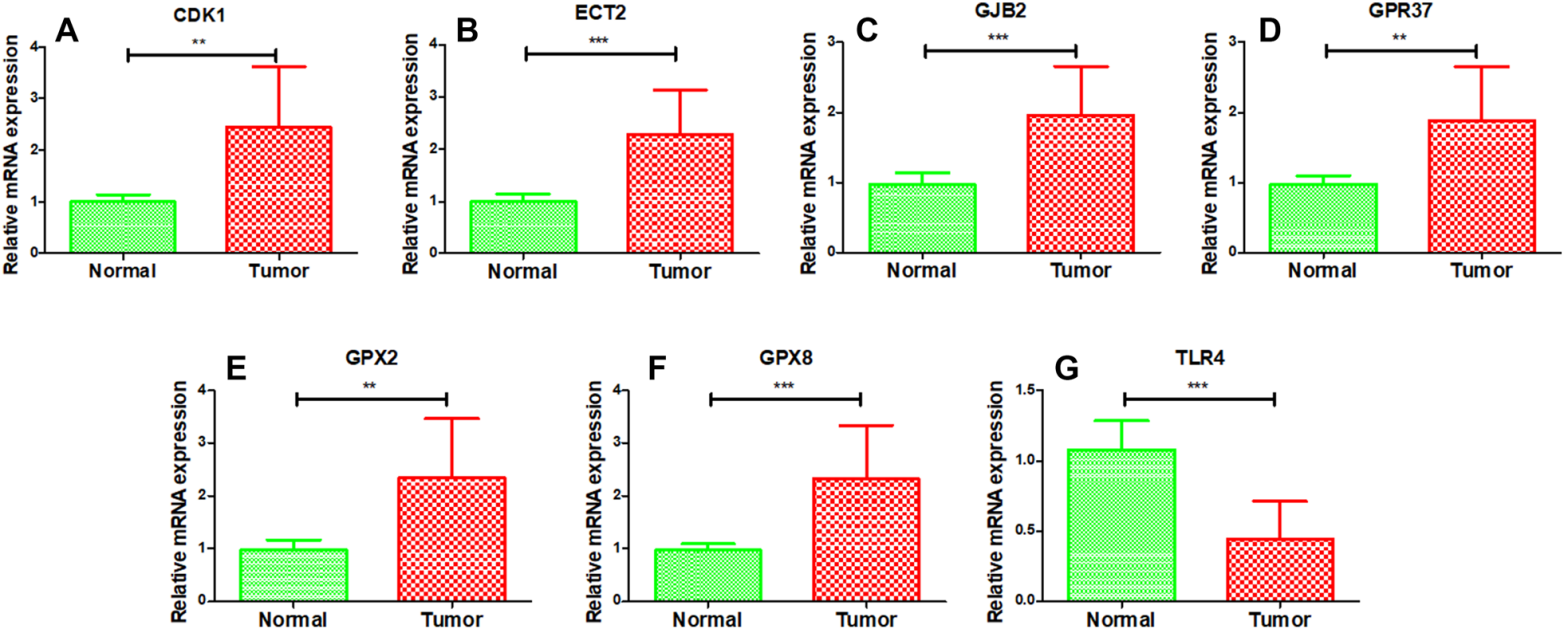
Validation of the expression of prognostic genes. The CDK1 (**A**), ECT2 (**B**), GJB2 (**C**), GPR37 (**D**), GPX2 (**E**), GPX8 (**F**), and TLR4 (**G**) gene expression levels were validated using clinical samples. *p < 0.05, **p < 0.01, ***p < 0.001. *T* test was used to analyze the differences between the two groups

In the present study, we collected clinical samples to validate the expression of these prognostic genes. The expression of CDK1, ECT2, GJB2, GPR37, GPX2, and GPX8 gene was up-regulated in the tumor group (*p* < 0.001), whereas the expression of TLR4 was down-regulated in the tumor group (*p* < 0.001) when compared with those in the normal group (Fig. 11). These findings were consistent with the bioinformatics analysis results.

## Discussion

LUAD is the most common subtype of lung cancer, lots of scholars have focused on the occurrence, progression, prognosis, and treatment of LUAD in recent years [36–38]. A growing number of reports have revealed that different subtypes of lung cancer exhibited different clinical outcomes and characteristics [39–41]; therefore, it is important to classify LUAD and personalized therapeutic interventions. The imbalance between the antioxidant defense system and reactive-oxygen species causes oxidative stress. Oxidative stress has been increasingly considered an important contributor to aging, and various forms of etiopathogenesis are commonly associated with aging [42]. Meanwhile, oxidative stress could also contribute to a variety of chronic diseases, including cancer, Alzheimer’s disease, atherosclerosis, etc. [43]. In addition, oxidative stress-related research has attracted the attention of many tumor fields. For example, reactive-oxygen species-mediated oxidative stress plays an important role in the pathogenesis of breast cancer through epigenetic/genetic mutations, which lead to uncontrolled cell proliferation [44]. Oxidative stress-driven autophagy plays a vital role in the onset and development of hepatocellular carcinoma [45]. 13 oxidative stress-related genes were associated with the development of gastric cancer [16]. The colorectal cancer-integrated oxidative stress score showed better predictive performance in colorectal cancer patients compared to the TNM stage [46]. However, the role of oxidative stress in the progression of LUAD is still poorly studied, and the research on the pathological mechanisms and prognostic markers of LUAD patients associated with oxidative stress-related genes is still scarce. Therefore, we aimed to develop a risk score model based on oxidative stress-related genes.

In the present study, 40 DEOSRGs were identified from TCGA-LUAD and GSE31210 datasets to investigate the prognostic value of oxidative stress-related genes. In addition, we revealed that the oxidative stress-related subtypes were closely associated with the prognosis and tumor immune microenvironment of LUAD. There is compelling evidence indicating that oxidative stress plays a critical role in causing an imbalanced immune system [47]. A recent study reveals that reactive-oxygen species serve dual roles in tumor development, acting not only as mediators of oxidative stress, but also as active participants in immune regulation [48]. Furthermore, chronic inflammation, caused by excessive production of reactive-oxygen species, is a contributing environmental factor that aids in tumor immunosuppression [49].

Next, we performed GSEA and GSVA to investigate the biological mechanisms underlying the two subtypes. The GSEA and GSVA results showed that the C1 subtype exhibited activation of pathways related to oxidative stress, inflammatory response, and immune response. The consensus among experts is that the tumor microenvironment is a long-term inflammatory setting that plays a crucial role in the formation and advancement of most tumors. Increasing evidence suggests that mitochondrial reactive-oxygen species have a key role in the inflammatory tumor microenvironment, ultimately exacerbating the growth of cancer [50, 51]. A research has demonstrated that oxidative stress not only influences the proliferation of cancer cells but also plays a pivotal role in modifying the immune microenvironment [52, 53]. The function of Tregs in the tumor microenvironment has been extensively studied, however, recent findings highlight a new mechanism of immune suppression that is associated with oxidative stress. In their investigation, Maj et al. examined the involvement of oxidative stress in inducing apoptosis of Treg cells within the tumor microenvironment [54]. A high level of reactive-oxygen species can suppress T cell responses by hindering the formation of the MHC and TCR antigen complex. This action allows cancer cells to evade immune responses and facilitates cancer progression [55]. In addition, the activation of the mitochondrial Lon-induced mtROS-NF-κB pathway triggers the release of inflammatory cytokines from cancer cells, leading to the establishment of immune suppression in the tumor microenvironment [56]. In summary, these results indicated that oxidative stress can regulate the infiltration of immune cells via diverse immune signaling pathways, ultimately leading to enhanced prognosis for individuals with LUAD.

To evaluate the predictive value of DEOSRGs in determining the prognosis of LUAD, 10 DEOSRGs were selected to develop a risk score model to predict the overall survival of LUAD patients. Interestingly, previous studies have reported these genes to play important roles in cancer progression: up-regulation of EZH2 was related to poor prognosis for lung cancer patients, accompanied by potential damage of viability and migration in lung cancer cells [57]. ECT2 accelerated LUAD development through focal adhesion signaling pathways and extracellular matrix dynamics [58]. HYAL1 inhibited colorectal cancer metastasis via the regulation of TIMPs/MMPs balance, further suppressing migration and invasion of colon cancer cells [59]. TLR4 expression was associated with poor prognosis in patients with nonsmall cell lung cancer [60]. GPX8 could impact the prognosis and tumorigenesis of nonsmall cell lung cancer patients via the regulation of epithelial characteristics [61]. GJB2 was associated with early-stage breast cancer development through the regulation of cancer stemness [62]. CDK1 positively regulated the lung cancer cell’s stemness via interacting with Sox2 [63]. Downregulation of GPR37 significantly suppressed the migration and proliferation of LUAD [64]. GPX2 contributed to malignant development and cisplatin resistance of Kirsten rat sarcoma viral oncogene homolog-driven lung tumorigenesis [65]. GPX3 inhibited lung cancer cell proliferation by regulating redox-mediated signals [66]. Although the regulation effects of these DEOSRGs had been investigated in various cancers, few researchers have systematically assessed their prognostic values in LUAD. Subsequently, multivariate Cox regression analysis revealed that the risk score model was an autocephalous prognostic predictor for LUAD patients. In addition, a predictive nomogram implied a better prediction of overall survival at 3- and 5-year compared with the ideal model. Overall, we first investigated the significance of “response to oxidative stress” for the prognosis of LUAD patients and were the first to develop a risk score model based on oxidative stress-related genes.

Another interesting finding of our study indicated that the oxidative stress-related risk score was significantly associated with immune cell infiltration, which further revealed the fact that oxidative stress is involved in the tumor immune microenvironment: oxidative stress could control regulatory T cells behavior and impact the efficacy of targeting cancer immune checkpoints [54]. Generation and regulation of reactive-oxygen species levels in tumor immune microenvironment-related cancer and stromal cells play a decisive role in cancer progression [52, 53].

## Conclusions

Two molecular subtypes were identified in LUAD based on the DEOSRGs through consensus clustering. These subtypes showed distinct survival times and immune statuses. Furthermore, a prognostic model related to oxidative stress was developed and validated, enabling a comprehensive study of LUAD progression and a deeper understanding of its underlying mechanisms.

### Supplementary Information

**Additional file 1: Figure S1.** The Kaplan-Meier survival curves of the macrophages (**A**), macrophages M1 (**B**), Tgd cells (**C**), pro B cells (**D**), Th1 cells (**E**), and Th2 cells (**F**). **Figure S2**. The CDK1 (**A**), ECT2 (**B**), EZH2 (**C**), GJB2 (**D**), GPR37 (**E**), GPX2 (**F**), GPX3 (**G**), GPX8 (**H**), HYAL1 (**I**) and TLR4 (**G**) gene expression levels were analyzed in TCGA database. *p < 0.05, **p < 0.01, ***p < 0.001. *T*-test was used to analyze the differences between the two groups. **Figure S3.** The Kaplan-Meier survival curves of the CDK1 (**A**), ECT2 (**B**), EZH2 (**C**), GJB2 (**D**), GPR37 (E), GPX2 (**F**), GPX3 (G), GPX8 (**H**), HYAL1 (**I**) and TLR4 (**G**) in LUAD.** Table S1**. LUAD patient characteristics. **Table S2**. Sequences of primers used quantitative real-time PCR.

**Additional file 2: Table S3.** DEGs list in GSE31210 dataset.

**Additional file 3: Table S4.** DEGs list in the TCGA-LUAD dataset.

## Data Availability

The data come from the TCGA database (https://portal.gdc.cancer.gov/) and the GEO database (https://www.ncbi.nlm.nih.gov/geo).
