# Supplementary material for: Identification of molecular subgroups and establishment of risk model based on the response to oxidative stress to predict overall survival of patients with lung adenocarcinoma
Source: Eur J Med Res. 2023 Sep 9;28:333. doi: 10.1186/s40001-023-01290-5 (PMC10492289; doi:10.1186/s40001-023-01290-5)
Supplement: Supplementary file 1 — Additional file 1: Figure S1. The Kaplan-Meier survival curves of the macrophages (A), macrophages M1 (B), Tgd cells (C), pro B cells (D), Th1 cells (E), and Th2 cells (F). Figure S2. The CDK1 (A), ECT2 (B), EZH2 (C), GJB2 (D), GPR37 (E), GPX2 (F), GPX3 (G), GPX8 (H), HYAL1 (I) and TLR4 (G) gene expression levels were analyzed in TCGA database. *p < 0.05, **p < 0.01, ***p < 0.001. T-test was used to analyze the differences between the two groups. Figure S3. The Kaplan-Meier survival curves of the CDK1 (A), ECT2 (B), EZH2 (C), GJB2 (D), GPR37 (E), GPX2 (F), GPX3 (G), GPX8 (H), HYAL1 (I) and TLR4 (G) in LUAD. Table S1. LUAD patient characteristics. Table S2. Sequences of primers used quantitative real-time PCR. [file 40001_2023_1290_MOESM1_ESM.docx]

Table S1 LUAD patient characteristics.

| characteristics | No. of cases |
| --- | --- |
| n | 10 |
| Gender, n (%) |  |
| Female | 2 (20%) |
| Male | 8 (80%) |
| Age, n (%) |  |
| <= 65 | 3 (30%) |
| > 65 | 7 (70%) |
| Smoker, n (%) |  |
| No | 3 (30%) |
| Yes | 7 (70%) |

Table S2 Sequences of primers used quantitative real-time PCR.

| Gene | Forward primer (5' to 3') | Reverse primer (5' to 3') |
| --- | --- | --- |
| CDK1 | GCGGAATAATAAGCCGGGAT | CAACTCCATAGGTACCTTCTCCA |
| ECT2 | GCTGTATTGTACGAGTATGCT | GTCACCAATTTGACAAGCTC |
| GJB2 | GGGCAATGCTTAAACTGGC | TATGACACTCCCCAGCACAG |
| GPR37 | GGCCAACAGTCTCAGATCAT | GTACTTCCAATCACAACTCAAACAC |
| GPX2 | GGTAGATTTCAATACGTTCCGGG | TGACAGTTCTCCTGATGTCCAAA |
| GPX8 | GTTTCACTAGTTGTAAACGTGGC | CGATTCTCCAAACTGATTGCAGG |
| TLR4 | ATGCCCCATCTTCAATTGTC | AGTGAGGATGATGCCAGGAT |
| GAPDH | ACCACAGTCCATGCCATCAC | TCCACCACCCTGTTGCTGTA |


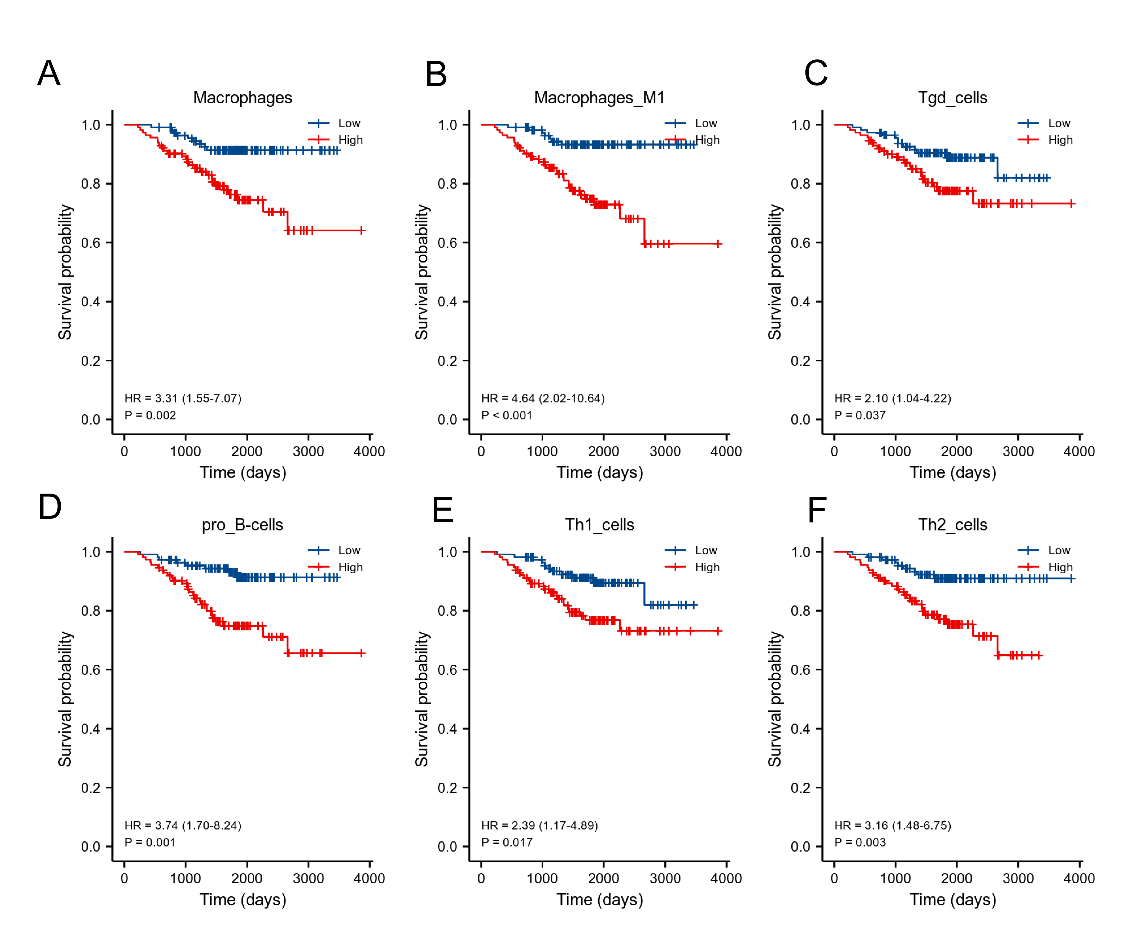


Figure S1 The Kaplan-Meier survival curves of the macrophages (A), macrophages M1 (B), Tgd cells (C), pro B cells (D), Th1 cells (E), and Th2 cells (F).


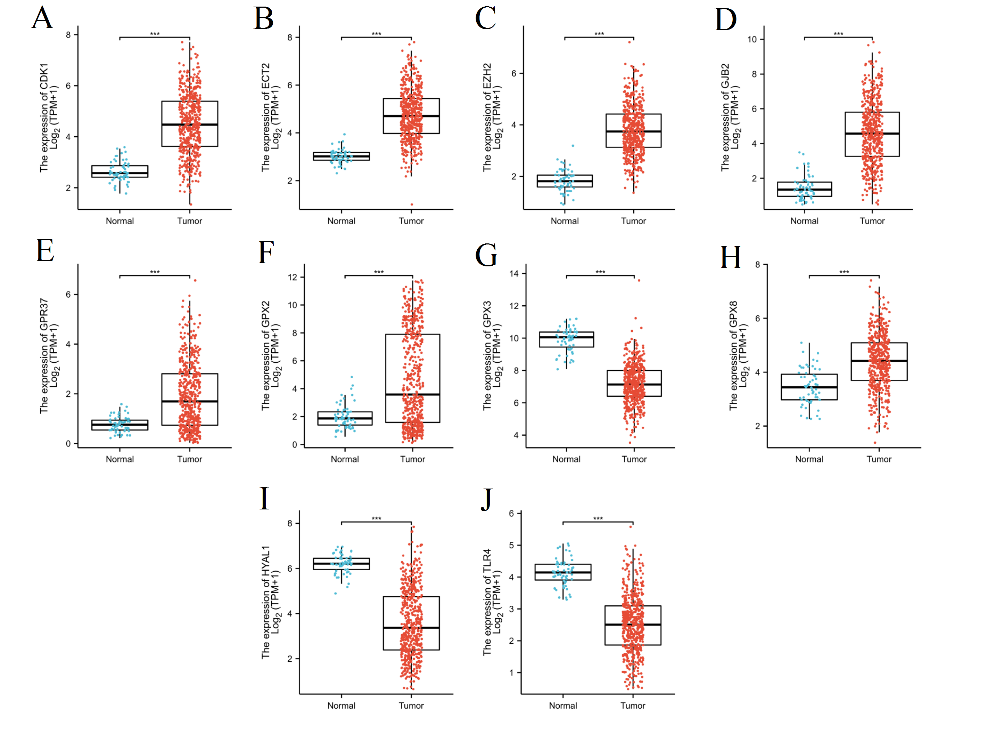


Figure S2 The CDK1 (A), ECT2 (B), EZH2 (C), GJB2 (D), GPR37 (E), GPX2 (F), GPX3 (G), GPX8 (H), HYAL1 (I) and TLR4 (G) gene expression levels were analyzed in TCGA database. *p < 0.05, **p < 0.01, ***p < 0.001. T-test was used to analyze the differences between the two groups.


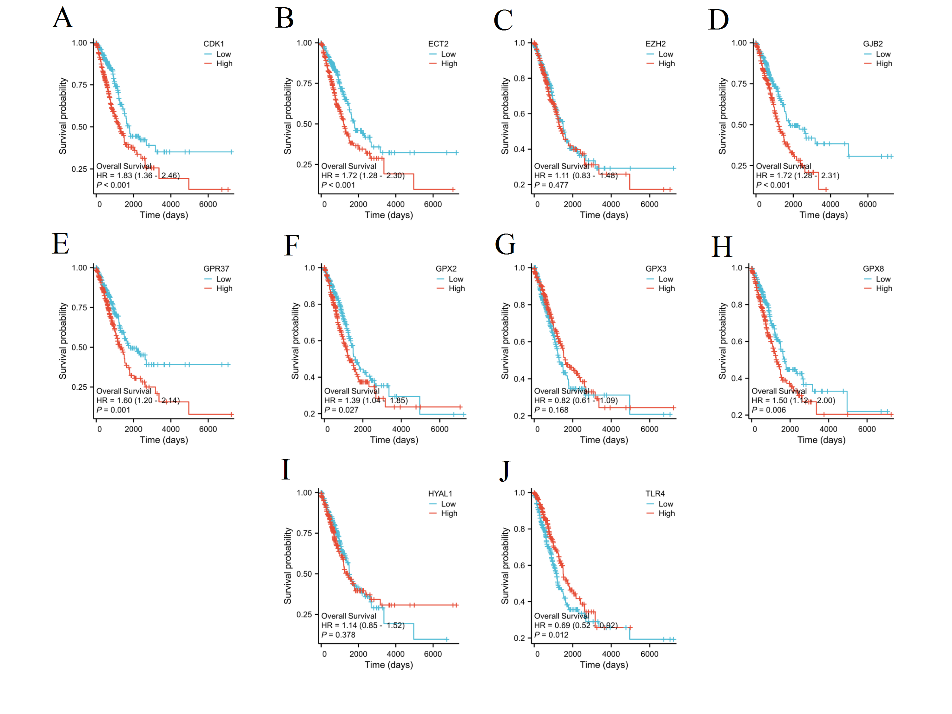


Figure S3 The Kaplan-Meier survival curves of the CDK1 (A), ECT2 (B), EZH2 (C), GJB2 (D), GPR37 (E), GPX2 (F), GPX3 (G), GPX8 (H), HYAL1 (I) and TLR4 (G) in LUAD.
